# Letters to Physicians

**Published:** 1912-01

**Authors:** George M. Gould

**Affiliations:** Atlantic City, N. J.


### Letters to Physicians

### Denutrition, "Migraine," Esophoria, Fading-Image and Other Diseases Caused by Eye Strain

By GEORGE M. GOULD, M.D.
Atlantic City, N. J.

ABOUT two years ago Mrs. G., up to the time of consulting me, had had sickheadaches with vomiting and nausea so long that she could not remember when they began. There had also been great dizziness with frequent weakness, as if she were about to fall. There were often "rushes of blood to the head," much indigestion, day-time drowsiness, "hurting of the head," feeling of dulness and stupidity, pain in the back of the neck, and although "tired all the time" she was constantly "nervous, tense and unable to sit still." Of course she had constant tilting of the head to one side. I found also that she had 20° of esophoria, with typic "fading image," the right in about three seconds, the left in about six seconds. And the retinas were so scarlet and stippled as to arouse great fear. There was venous pulse upon both discs. The woman was very much emaciated. A permanent cycloplegia for three weeks was at once ordered. One of her several former physicians had told her when she spoke of eyes, that her sick headaches were "due to other causes." She had taken quantities of medicines of many kinds, and her last oculist had ordered B.E.+Cyl.0.25 ax. 90°, a correction of her ametropia as accurate and effective as would have been that of any medieval charm. Her sick headaches, pains and mental and nervous troubles disappeared immediately with the first atropin drops that were instilled. Her static refraction was found to be:

R.+S.1.50 +C.O.25 ax.90°=20|30)

L.+S.1.37 +C.O.37 ax.90°=20|30(

When the effect of the drops passed out of her eyes there was a great feeling of discomfort experienced but the old-time symptoms did not recur. "Has not felt so well in five years." When she first came she could hardly walk from the street car to the house; even four months afterward the "fading image" still persisted, and there was inability to read without signs of ill-health. In the four months she had gained 20 lbs. in weight. Fifteen months later she lost 10 of her 20 lbs. gained, dull headaches began to come back, with some "stomach trouble," etc. A change in the refraction-error was found which accounted for the relapsing tendency.

This case, illustrative, in most respects, of an enormously large number, suggests certain queries:

1. In an unknown but high proportion of all cases in which systemic reflexes and effects of eyestrain exist, there is proof beyond question that eyestrain causes denutrition, often reducing the sufferers to under-weight, and even to positive emaciation. Why is this proof, and the fact it proves, so utterly neglected?

2. "Migraine," or sick headache in my experience is absolutely always the result of ametropia, uncorrected or malcorrected. Why are the millions of sufferers from this hideous disease allowed to continue in their suffering, when every case may be prevented and cured?

3. The most frequent and disastrous of the local inflammations of the eyes, such as retinitis, choroiditis, glaucoma, and cataract, rarely ever—one might say, never—arise in eyes which have had their ametropias adequately corrected. Why is this truth, revolutionizing ophthalmology, almost universally denied, or ignored?

4. Heterophorias are the results of ametropia. Why do so many of the oculists of the world believe and treat them as causes of eyestrain, and by decentering lenses, or by tenotomizing, or by not preventing the ametropic effects, through correction of the ametropia, keep up the farce of a specialty almost every one of the members of which disagrees with the others in such a highly important phase of practice?

5. Why is a most incapaciating and significant symptom, "Fading Image" and the disease I have named, "Ophthalmovascular Choke" universally ignored in ophthalmologic practice and reports?

The answer to these questions is obvious: Because the differential diagnosis by means of cycloplegia is never tried; and because there is no agreed-upon science of the practical diagnosis of errors of refraction. One hundred oculists would diagnose and treat a single patient in one hundred mutually exclusive and contradictory ways. Not a hint exists that this miserable scorn-provoking condition should be ended by the establishment of a scientific School of Refraction which would finally extinguish this egregious professional disgrace.

